# The Influence of Vegetable Oil Addition Levels on the Fatty Acid Profile and Oxidative Transformation Dynamics in Liver Sausage-Type Processed Meats

**DOI:** 10.3390/foods14030380

**Published:** 2025-01-24

**Authors:** Agnieszka Bilska, Mirosława Krzywdzińska-Bartkowiak

**Affiliations:** Department of Meat Technology, Poznan University of Life Sciences, Wojska Polskiego 31/33, 60-624 Poznan, Poland; miroslawa.krzywdzinska-bartkowiak@up.poznan.pl

**Keywords:** fatty acids, liver pâté, sensory evaluation, vegetable oils, texture analysis

## Abstract

In the production of meat products, animal fats, which are rich mainly in saturated fatty acids, are used as a recipe ingredient. To improve the quality and fatty acid profile of meat products, it is possible to partially replace animal fat with vegetable oils. This approach aims to achieve a more favorable PUFA/SFA ratio and n-6:n-3 PUFA ratio, bringing them closer to the values recommended by nutritional organizations. Therefore, the aim of this study was to determine the impact of replacing 20% and 40% of animal fat with selected plant fats on the change in the fat fraction composition of liver pâté-type processed meat and its oxidative stability. Fatty acid content was analyzed in the oils purchased from retailers and in experimental samples. During refrigerated storage of the experimental sausages, changes in the content of primary (peroxide value (PV)) and secondary oxidation products (TBARS), as well as changes in sensory quality, were evaluated. The analysis included cross-sectional color, aroma, texture, saltiness, and taste. The study showed that replacing 20% of animal fat with vegetable oils resulted in products with high sensory attractiveness and oxidative stability, outperforming those with 40% replacement. Among the tested vegetable oils, samples with rapeseed oil demonstrated the highest oxidative stability and the most favorable, nutrition-recommendation-approaching n-6 to n-3 fatty acid ratio, compared with samples with flaxseed, corn, sunflower, and soybean oils.

## 1. Introduction

Liver sausages are consumed worldwide and are popular among a considerable group of consumers. They are produced from various types of meat and fat. They typically contain large amounts of animal fat (about 35%), which is a source of saturated fatty acids, constituting about 50% of all acids. Due to their high fat and cholesterol content, dietitians advise against excessive consumption of liver sausages, although these products can be treated as a valuable source of vitamin A and non-heme iron (about 30 mg/g of the sample) [1,2,3], with iron being considered the most important pro-oxidant in meat products. Liver pâtés are products with high oxidative instability. Additionally, the cutting/mixing of ingredients during the production process increases oxidative instability, causing interactions between free fatty acids and oxygen in the presence of catalysts such as heat and metalloproteins [4,5].

There is a current trend to improve the nutritional value of meat products with a high fat content by reducing the amount of fat. This may lead to undesirable technological and sensory changes in the meat product [6,7,8]. Fatty raw products are of great functional importance, such as by forming the texture, contributing juiciness and conveying flavor. Therefore, reducing the fat content of a meat product can adversely affect its palatability, juiciness and texture [9]. An alternative method to reduce the content of saturated fatty acids is to eliminate some of the raw fat from the recipe and replace it with vegetable oils which contain less saturated fatty acids (SFAs) and are richer in monounsaturated fatty acids (MUFAs) and polyunsaturated fatty acids (PUFAs) [10].

The chemical composition of vegetable oils is relatively diverse and changes depending on many factors. The most important are the type and variety of the raw material from which the oil is obtained, its maturity, as well as weather and agrotechnical conditions occurring during cultivation. The quality of the raw material, the method of oil production and the method of further handling of the oil, including purification methods, are also of great importance. In addition, the method of packaging the oil and its storage conditions should also be mentioned. The source of vegetable oils are primarily the seeds of cultivated plants, such as rapeseed, common sunflower, soybean, olives, common flax, corn, poppy, cotton, coconut or oil palm. These oils are characterized by a high content of unsaturated fatty acids, such as linolenic, linoleic or oleic acid, with the proportions of these acids dependent on the type of oil. Most vegetable oils remain in a liquid state at room temperature, with the exception of coconut and palm oil, which are solids in such conditions. Vegetable oils are a mixture of various fatty acids, carotenoids, phospholipids and natural antioxidants. Vegetable oils and fats therefore play an important role in the diet, contributing to the proper functioning of the body, providing energy, EFAs, vitamins (A, D, E and K) and other bioactive compounds.

Rapeseed oil, called the olive oil of the north, contains about 10% ALA (C18:3, linolenic acid), 1% linoleic acid and 57% oleic acid. Rapeseed oil, in addition to unsaturated fatty acids, contains nine functional components that contribute to its health benefits, including anti-microbial, anti-inflammatory, anti-obesity, antidiabetic, anti-cancer, neuroprotective, and cardioprotective effects. These components are as follows: vitamin E, flavonoids, squalene, carotenoids, glucoraphanin, indole-3-carbinol, sterols, phospholipids, and ferulic acid. Each of these substances, either in their original form or as derivatives, possess beneficial health properties [11,12,13].

Linseed oil is characterized by a content of linolenic acid at a level of 30–60%, linoleic acid 10–25%, oleic acid 13–26% and palmitic acid 5.4–9.4%. Other components of linseed oil (0.1–0.9%) include, among others, natural dyes such as chlorophyll (approx. 0.4 mg/kg), carotenoids (approx. 147.5 mg/kg), tocopherols (alpha—0.6 mg/100 g, gamma—72.1 mg/100 g, delta—1.7 mg/100 g), glycerophosphates (lecithins and cephalins), hydrocarbons, waxes, steroids, enzymes, essential oils, phosphates and phospholipids. The content of vitamin E in the amount of approx. 25 mg/100 g of linseed oil constitutes 250% of the daily requirement for this vitamin [14,15].

Soybean oil contains approx. 6–7% linolenic acid. However, linoleic acid present in soybean oil is subject to oxidative processes, which makes this oil less durable due to the formation of, among other things, fragrances. In addition to fatty acids, lipophilic components of vegetable oils that exhibit E-vitamin activity (tocopherols) and lower cholesterol levels (phytosterols) also deserve special attention. Tocopherols are also compounds that protect the body against excess free radicals and other reactive forms of oxygen [16,17].

Corn oil is characterized by a high content of polyunsaturated fatty acids, including linoleic acid (45–70%), oleic acid (30%), palmitic acid (13%) and stearic acid (4%). It is a good source of carotene and tocopherol. These components have an impact on reducing oxidative stress in cells and cholesterol levels. The disadvantage of corn oil is its ratio of omega-6 to omega-3 acids, which is 59:1 [13,18,19,20].

Sunflower oil is a versatile and healthy oil, rich in unsaturated fatty acids—it usually contains around 50 to 75% linoleic acid and 10 to 40% oleic acid. It is a rich source of vitamin E (30 mg/100 g), especially in the form of gamma-tocopherol, which has antioxidant properties that protect cells from damage caused by free radicals. Sunflower oil contains plant sterols and small amounts of phospholipids such as lecithin [21,22,23]. There are different varieties of sunflower oil, depending on the balance of fatty acids and the extraction method: high linoleic sunflower oil, which contains higher amounts of polyunsaturated fatty acids, especially linoleic acid, making it more susceptible to oxidation, but beneficial to health in moderation; high oleic sunflower oil, which contains more monounsaturated oleic acid, is considered more stable and healthier due to its lower susceptibility to oxidation, has a longer shelf life, and is used in a variety of food products, including those requiring frying; and medium oleic sunflower oil, which is a balance between high linoleic and high oleic, offering a moderate amount of monounsaturated and polyunsaturated fats [22,23,24].

The aim of the study was to examine the impact of partially replacing animal fat with vegetable oils on the quality of liver pate. Particular attention was given to analyzing changes in the proportions of saturated and unsaturated fatty acids resulting from this recipe modification. The research also included an assessment of the product’s oxidative stability, texture-forming properties, and sensory parameters such as taste, aroma, consistency, and overall consumer acceptance.

## 2. Materials and Methods

### 2.1. Material

The oils used to produce the experimental sausages [corn (CO), linseed (LO), rapeseed (RO), sunflower (SunO) and soybean (SO)] were purchased from a retailer (Poznan, Poland).

The material studied was offal sausages of the pâté type. The meat raw materials used for the production of experimental sausages were purchased from a local butcher shop (Poznan, Poland). The following spice mixture was added during chopping (per 1 kg of batter): 1.5% salt, 0.15% pepper, 0.05% marjoram, and 0.4% onion. This composition served as the reference product for the experimental variants.

A range of sausages was also produced in which some of the animal fat was replaced by corn (CO), linseed (LO), rapeseed (RO), sunflower (SunO) and soybean (SO) oils. This oil accounted for 20% and 40% of the fine animal fat (Table 1).

The production process of the experimental sausages was as follows: the meat fat raw material was cooked until semi-soft, then subjected to the chopping process, with the prescribed oil, broth, and spices added. Towards the end of the chopping process, pre-chopped liver was added. The final temperature of the batter was 40 °C.

The batter was used to fill semi-permeable collagen casings with a diameter of 40 mm. After filling, the offal sausages of the pâté type were scalded until a temperature of 70 °C was reached in the geometric center of the baton, and then cooled to a temperature of 4 °C.

After cooling, the finished products were placed in a cold store at a temperature of approximately 4 °C. For testing, samples were taken on the 1st, 5th, 8th, 12th and 15th days after production.

### 2.2. Methods

#### 2.2.1. Determination of Basic Chemical Composition

Water content was determined following ISO 1442:1997 [25]. Total protein content was measured using the Kjeldahl method according to ISO 5983-2:2009 [26] utilizing the Kjeltec-2200 system (Foss, Hilleroed, Denmark). Fat content was analyzed by the Soxhlet method following ISO 3960:2010 [27] using the Soxtec-HT6 system (Foss) and the chloride content was analyzed using the Volhard method [28].

#### 2.2.2. Determination of the Fatty Acid Profile

The fat from the experimental sausages was extracted according to the procedure described by Folch et al. [29]. A chloroform: methanol mixture (2:1 *v*/*v*) was used.

The fatty acid composition was determined following the AOCS Official Method Ce 1 h-05 [30]. Oil samples (10 mg) were dissolved in hexane and transesterified with sodium methylate (0.1 M). Fatty acid methyl esters (FAMEs) were analyzed using an Agilent 7820 A GC (Agilent Technologies, Santa Clara, CA, USA) equipped with SLB-IL111 capillary columns (Supelco, Bellefonte, PA, USA; 100 m, 0.25 mm, 0.20 μm) and a flame ionization detector (FID). The oven temperature was initially set to 150 °C and increased to 200 °C at a rate of 1.5 °C/min. The injector and detector temperatures were both 250 °C, with a split ratio of 1:10. Helium was used as the carrier gas at a flow rate of 1 mL/min. FAMEs were identified by comparison with commercially available standards—grain fatty acid methyl ester mix (Supelco, Bellefonte, PA, USA). Results were expressed as percentages of the total fatty acids.

#### 2.2.3. Storage Tests

- Determination of primary and secondary lipid oxidation products

Peroxide value (PV)

The peroxide value (PV) was determined according to the ISO standard [27] in the extracted lipid fraction, following the procedure of Folch et al. [29] (using a chloroform solvent system at a ratio of 2:1 (*v*/*v*). Results were expressed in milliequivalents of O_2_ per kilogram (meq. O_2_ * kg^−1^).

Malondialdehyde content measured by TBARS method

The 2-thiobarbituric acid reactive substances (TBARS) value was determined using the distillation method of Tarladgis et al. [31] as modified by Pikul et al. [32]. Results were expressed as milligrams of malondialdehyde (MDA) per kilogram (mg MDA * kg^−1^).

- Sensory evaluation of quality

Sensory evaluation was prepared and carried out according to the guidelines contained in the following standards: PN-EN-ISO13299:2016 and PN-EN-ISO5492:2009 [33,34].

A 5-point scale method was employed for the sensory evaluation of the experimental sausages. The liver pâtés were assessed at room temperature. Each sample was sliced into approximately 2 cm thick portions, which were arranged on white plates and labeled with unique identification numbers. The samples were evaluated for taste, aroma, color, texture, perceived greasiness, and overall desirability. Specific attributes assessed included cross-sectional color, aroma, texture, saltiness, and flavor, with a scale ranging from 1 (extremely dislike) to 5 (extremely like).

Overall desirability was calculated using the following weighted factors: 0.3 for cross-sectional attributes and 0.7 for oral characteristics. The evaluation was conducted by a panel of 16 trained assessors who had undergone prior instruction in sensory analysis methodology.

#### 2.2.4. Texture Analysis

For the texture analysis of the pates, a TA.XTplus texture analyzer (Stable Microsystem, Godalming, UK) equipped with a measuring head with a maximum pressure force of 5 kg and a conical plexiglass sensor designed for spreadability testing (HDP/SR, Stable Microsystem, Godalming, UK) was used. The hardness (maximum force required to penetrate the sample in N), shear work (spreadability) [N × s] (the area under the curve), stickiness as the peak force in the range of negative values [N], and work of adhesion [N × s] as the area above the time–force curve were measured. The textures of the pates were tested after 15 days of storage.

### 2.3. Statistical Analysis

The results were statistically analyzed with the use of STATISTICA 13.3 and Excel 2010. The results presented in this paper are the arithmetic average of two experimental series and three repetitions. Comparisons of mean values of the studied traits were made using analysis of variance for factor systems, and intergroup differences were assessed using the Tukey test. Statistical inference was performed at a significance level of *p* ≤ 0.05.

The study of interdependence between variables was performed using linear regression analysis y = Ax + B, where y—dependent variable (value of the parameter under study), x—independent variable (type of sample, storage time), A—coefficient on the independent variable (tg of the angle of slope of the curve), and B—constant term. Statistical analysis of changes in the regression angle coefficient (coefficient of A/24 h) allowed the dynamics of the changes to be determined.

## 3. Results

### 3.1. Determination of Fatty Acid Composition Content in Selected Edible Oils

In this study, vegetable oils with different chemical compositions were selected for the experiments. Corn, linseed, rapeseed, sunflower and soybean oils were selected. The results of the individual fatty acid content of the selected edible oils and animal fat (so-called fine fat) are presented in Table 2. They differed in their fatty acid composition, as well as in the amount of saturated, monounsaturated and polyunsaturated fatty acids. The highest content of saturated fatty acids was identified in animal fat. These were palmitic (C16:0), stearic (C18:0), lauric (C12:0) and myristic (C14:0) acids, which were not detected in the vegetable oils tested. Among the vegetable oils, corn and soybean oil had the highest saturated acid content. Rapeseed oil contained the highest amount of monounsaturated fatty acids, including 61.95% of oleic acid, while the other oils studied contained less than half this amount.. Sunflower oil (63.91%), as well as soybean oil (56.84%) and corn oil (55.65%), were the richest sources of linoleic acid (C18:2). The main representative of polyunsaturated fatty acid that contain a double bond at the n:3 position is α -linolenic acid (C18:3). Among the oils tested, the highest amount was found in linseed oil (51.82%).

Therefore, the consumption of these oils should be limited as part of a healthy diet. Among the oils tested, rapeseed oil was characterized by the lowest content of ∑SFA (6.88%), while the highest content of these acids was found in corn oil (15.70%) and soybean oil (15.25%). The levels of unsaturated fatty acids in the oils tested were high compared with saturated acids. Rapeseed oil had the lowest amount of ∑PUFA (30.56%) and the highest amount of ∑MUFA (62.56%). The highest proportion of polyunsaturated acids in the total fatty acid pool was found in linseed oil (67.94%), sunflower oil (64.24%) but was slightly lower in soybean oil (60.88%) and corn oil (56.75%).

PUFA/SFA ratios in the edible oils tested ranged from 3.61 (CO) to 6.86 (LO). However, the very low PUFA/SFA value in animal fat (0.22) indicates the dominance of saturated acids.

The proportion of both acid families that was closest to the dietary recommendations was found in animal fat (4.75:1). Among the oils tested, the most similar value to the suggested ratio of n-6 to n-3 acids was found in rapeseed oil (1.7:1). In sunflower and corn oils, n-6 fatty acids were predominant.

### 3.2. Characteristics of the Chemical Composition of Experimental Sausages

On the first day of the study, the basic chemical composition of the experimental sausages was determined, i.e., the content of protein, fat, and water. According to the Polish Standard PN-A-82007:1996/Az1:1998, (1998) [35] the fat content in offal sausages should not exceed 60%, while the water and protein content is not standardized in these products. Based on the conducted research, it was found that the average fat content in the produced sausages was 37–39%, which met the normative requirements in this regard. Based on the results of the fatty acid content determination in the studied offal sausages, it was found that the highest content of saturated fatty acids was in the control sample (Table 3). Replacing animal fat with oil in the amounts of 20% and 40% resulted in a reduction of these acids in the sausages. The highest reduction, about 13%, in SFA content was observed in samples with linseed oil.

According to the results summarized in Table 3, it can be observed that the samples with 20% and 40% replacement of animal fine fat with rapeseed oil were characterized by the highest MUFA, with lower PUFA content. This is related to an increase in the proportion of oleic acid of about 10% in these samples.

The experimental sausages were characterized by a low content of α-linolenic acid (ALA). Nevertheless, the most notable of these were samples in which rapeseed oil was used as a partial substitute for animal fat, where the content of this acid was on average nine times higher than in samples with corn or sunflower oil (Table 4). In contrast, sausages with animal fat substitution with linseed oil contained six times more ALA than in the rapeseed oil sample. It is also important to consider the results of the analysis of the proportions of n-6 to n-3 family acids. The samples with animal fat replaced by rapeseed oil were the most consistent with the dietary recommendations. The ratio of n-6/n-3 fatty acids in these samples ranged from 4.3:1 to 4.4:1. Unfavorable proportions of n-6 to n-3 family acids were observed in samples where fine animal fat was replaced with corn, sunflower and soybean oils (Table 4). The highest proportion of these acids was observed in samples involving fat substitution with corn oil; in the case of a 40% substitution, the proportion was as high as 123:1. However, in samples involving 20% and 40% substitution of animal fat for sunflower oil, the ratios were 74.4:1 and 89.9:1, respectively. Based on the results, a nearly two-fold increase of ∑PUFA in the total fatty acid pool was noted in the samples with substitution of animal fine fat by linseed oil, as compared with the control sample. The proportion of α-linolenic acid in this sample increased 10 fold. After 15 days of storage under refrigeration, the proportion of n-6/n-3 fatty acids in the experimental sausages did not alter. Furthermore, the most favorable proportion of acids of the n-6/n-3 family (4.7:1) was observed in the experimental sausages in which 20% of the fine animal fat was replaced with rapeseed oil.

### 3.3. Determination of Primary and Secondary Fat Oxidation Products in Experimental Sausages

Changes in the fraction of fat can be assessed by altering the content of individual fatty acids or by evaluating the content of their oxidation products. This second method is more useful for assessing the impact of these changes on the nutritional value of food products.

The oxidative changes in lipids in the tested products were evaluated by monitoring changes in the content of primary (peroxide value (PV)) and secondary oxidation products (TBARS) during refrigerated storage. Based on the statistical ANOVA analysis, it was concluded that the type of sample (the type and the amount of oil replaced) and the storage time had a statistically significant effect on the alterations of the selected oxidative fat quality parameters of the experimental sausages.

In the experimental sausages produced with the replacement of animal fat with corn, linseed, sunflower and soybean oils, the peroxide number value increased statistically significantly throughout the storage period (Table 5). The highest peroxide increase throughout the study occurred in the linseed oil sample, regardless of its level of replacement. A similar correlation was found in the experimental liver pâtés that were characterized by a high content of polyunsaturated fatty acids. These were samples involving the substitution of fine animal fat with corn, sunflower and soybean oil.

Based on the regression coefficients obtained (Table 5), it was found that the highest degree of inhibition of peroxide formation was observed in the control sample. Among the variants in which part of the fine animal fat was replaced with oil, it was observed that cold cuts with rapeseed oil were characterized by a statistically lower content of primary oxidation products compared with samples with corn, linseed, sunflower, or soybean oil. Among the analyzed cold meats, the samples with animal fat substituted by rapeseed oil had a significantly lower peroxide value during the entire period of storage. This most probably results from the lowest amount of polyunsaturated fatty acids in these samples.

The average results from the obtained tests are summarized in Table 6. On the first day of testing, the lowest TBARS content was detected in the sample in which 40% fat was substituted by rapeseed oil, and the highest in the control sample (1.18 mg MDA/kg and 1.99 mg MDA/kg, respectively). During the period of storage, TBARS content increased statistically significantly in most of the samples tested. The linseed oil sample had the highest values of TBARS throughout the storage period. The dynamics of the increase in secondary lipid oxidation products in the samples with animal fat substitution by corn, rapeseed, sunflower and soybean oils was lower in comparison with the control sample. However, the least dynamic changes in the TBARS index during the storage period were observed for samples involving 20% replacement of animal fat with rapeseed oil.

Analysis of the regression coefficients demonstrated that the sample with the substitution of animal fat by soybean oil in the proportion of 40% was characterized by the best oxidative stability among the experimental pâtés tested. The slope values for cold meats containing corn, linseed and sunflower oils were slightly higher compared with samples with rapeseed oil. It was observed that the TBARS index levels in the tested liver pâtés in which animal fat was replaced by corn, linseed, sunflower and soybean oils were lower or similar to the values observed in the control sample, regardless of the amount being replaced.

### 3.4. Texture Analysis of Experimental Sausages

Pâté usually has a spreadable consistency. When assessing spreadability, adhesion is of great importance because spreadability is the ease with which consumers spread a given product on bread. Adhesion is a desirable feature in the case of spreadable products and can be related to the ability to adhere to both the bread and the knife used for spreading. The closer the adhesion values are to zero, the worse the adhesion of the system. Adhesion close to zero is characteristic of liquids. This is undesirable in the case of systems intended for spreading. It must be noted that the higher the value of spreadability (work of shear), the lower the spreadability of the product.

Table 7 displays the impact of the replacement of animal fat by vegetable oils in pâtes. All experimental pâtés showed a significant decrease in firmness and shear work (*p* ≤ 0.05) with respect to those of the control sample. The same relationship was observed between all pâté samples with 20 and 40 percent replacement of animal fat with vegetable oil. However, in the case of sunflower and soy oil, these differences were not statistically significant.

It should be said that the use of vegetable oils as animal fat replacers in pates enhanced their spreadability. The experiment shows that replacing animal fat with vegetable oils from 20% to 40% reduces the firmness and shear work of pates, and thus increases spreadability. This means that the higher the share of oil in the pâté, the lower the solid phase content at a given temperature, and consequently the lower hardness and better spreadability.

### 3.5. Sensory Evaluation of the Quality of Experimental Sausages

The conducted studies also examined the impact of the type and degree of substitution of fine animal fat with vegetable oils on the sensory quality of the produced offal sausages of the liver pâté type. The produced sausages were evaluated using a 5-point sensory method. The evaluation criteria included cross-sectional color, aroma, texture, saltiness, and taste. Overall desirability was also calculated, taking into account the weighting factors.

For the consumer, the most important quality characteristics of the finished product, which can be directly recognized, are mainly color, smell and taste. The study revealed a statistically significant influence of the type of sample (i.e., the type and the amount of replaced oil) and the storage time on changes in the evaluated sensory quality characteristics. It was concluded that the samples in which 40% of the animal fat was substituted with vegetable oil obtained significantly lower scores in the evaluation of the tested quality characteristics in comparison with the variants with the 20% substitution. The greatest differences were observed in the evaluation of color in cross-section and texture of pâtés. The samples in which 40% of the animal fat was substituted with vegetable oil had lighter color and too loose a texture. Moreover, it was found that samples in which animal fat was substituted with corn, linseed and sunflower oils, regardless of the degree of substitution, had similar sensory quality in terms of smell and taste. On the other hand, the sample in which 20% of animal fat with rapeseed oil showed the highest stability of the studied descriptors during 15 days of storage.

The experimental sausages had a sodium chloride content of 1.60% in the control sample and 1.65–1.70% in the samples in which fine animal fat was substituted with vegetable oils. During storage, the saltiness of the test samples was assessed using the sensory method. It was found that the samples in which fine animal fat was substituted with vegetable oils, regardless of the level of substitution, had a more intense salty taste sensation compared with the control sample. Those samples received statistically significantly lower saltiness scores compared with the control variant. There was also a significant difference in terms of saltiness between samples in which 20% or 40% of animal fat was substituted with vegetable oil.

The desirability of the finished product’s taste was also assessed. The sensory evaluation found that the type of the aforementioned oil had a significant effect on the taste desirability of the experimental sausages. The sample in which 20% of animal fat was substituted with rapeseed oil had the most desirable taste compared with the other experimental sausages throughout the storage period. In contrast, the lowest scores were given to variants in which animal fat was substituted with soybean oil, regardless of the degree of substitution. Furthermore, there were no differences in terms of taste desirability between the sausages in which fine animal fat was substituted with corn oil and sunflower oil.

**Figure 1:**
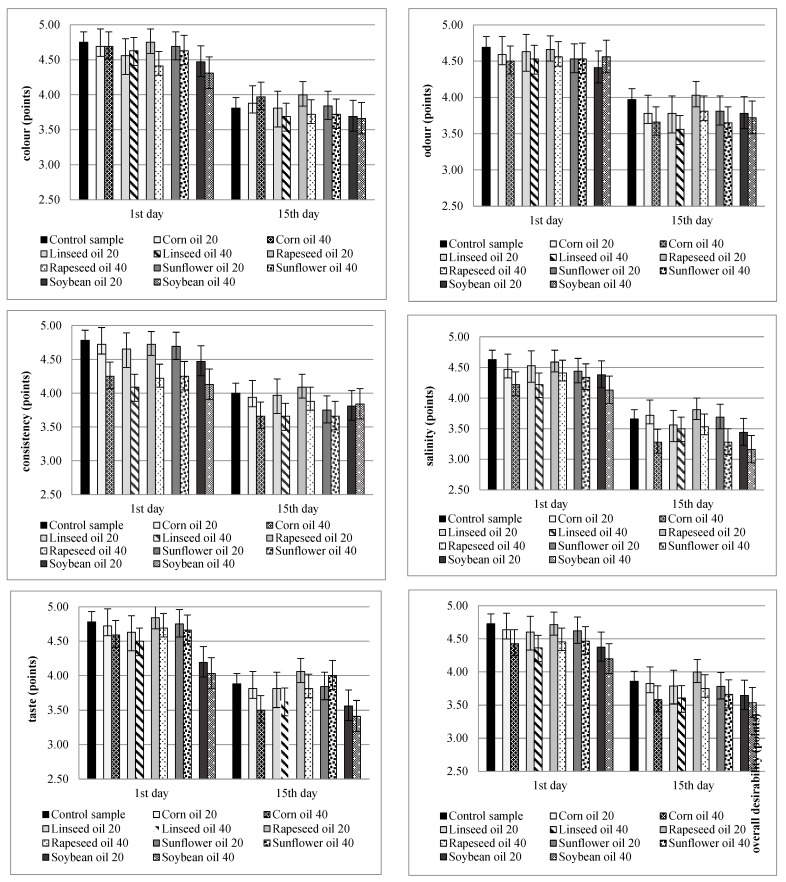
The sensory evaluation of experimental sausages on the 1st and 15th days after production $\bar{x}$ (*n* = 16) ± sd.

The overall desirability of the experimental sausages was calculated by considering the weighting factors. On the first day after production, the sample in which 40% of fine animal fat was substituted with soybean oil scored the lowest number of points (4.23 pts) while the control sample scored the highest number of points (4.73 pts). On day 15 after production, the sausage in which 40% of animal fat was substituted with soybean oil had the lowest average score. However, the sample in question had the least changes in overall desirability throughout the storage period. More pronounced differences can be observed in the simplified Figure 1, which includes limiting storage times. It was also observed that samples in which 40% of animal fat was substituted with vegetable oil had poorer overall desirability and received statistically significantly lower scores, when compared with samples with 20% substitution on the 1st and 15th days of testing.

## 4. Discussion

Vegetable oils differ in their fatty acid composition and the content of chemical compounds with antioxidant properties. In practice, various oils with different chemical compositions can be used, for example, to enrich products with unsaturated fatty acids. Olive oil and rapeseed oil are highest in oleic acid (C18:1). In contrast, soybean, corn, and sunflower oils contain between 55.07% and 65.90% linoleic acid (C18:2), which is more susceptible to oxidation than monounsaturated oleic acid [12,13]. In the conducted studies, rapeseed oil had the highest oleic acid content at 61.95%, while the other examined oils contained less than half that amount. Additionally, it was observed that sunflower oil (63.91%) was the richest source of linoleic acid (C18:2), followed by soybean oil (56.84%) and corn oil (55.65%)

The high content of unsaturated fatty acids in oils, especially linolenic acid, which is valuable from a nutritional point of view, means that these products are not very resistant to external factors such as oxygen, light or increased temperature [13,36]. Among the oils tested, the lowest amount of alpha-linolenic acid (C18:3) was found in sunflower oil (0.33%), while the highest amount was found in flaxseed oil (51.82%).

Saturated fatty acids primarily serve as a source of energy for the body. However, when consumed in larger quantities, they raise atherosclerotic lipoprotein levels in the blood and increase blood clotting [37,38]. Therefore, their consumption as part of a healthy diet should be limited. Animal fat had the highest content of saturated fatty acids. Among the tested oils, corn oil (15.70%) and soybean oil (15.25%) contained the most saturated fatty acids. In the studied oils, the content of saturated fatty acids was typical for the analyzed products. Similar levels of saturated fatty acids in sunflower, peanut, rapeseed, soybean, and corn oils were reported by Mińkowski et al. [39] and Awogbemi et al. [40].

Experts in the field of human nutrition recommend that the mutual proportion of n-6 to n-3 family acids in the diet should range from 4:1 to 6:1 [38,41,42,43]. However, it is also possible to find information that this ratio should be below 4:1 or even 1:1 [8,43,44]. The conducted studies found that animal fat had the most favorable ratio of fatty acids, closest to the recommended proportion (4.75:1). Among the oils tested, rapeseed oil showed the most favorable n-6 to n-3 fatty acid ratio, at 1.7:1. In contrast, sunflower and corn oils were dominated by n-6 fatty acids. The n-6 to n-3 ratio in these oils was 191.4:1 and 50.59:1, respectively.

Among unsaturated fatty acids, MUFAs have a more beneficial effect because, unlike PUFAs, they do not reduce the concentration of high-density lipoproteins (HDL) in the blood, which protect against coronary heart disease [38,45]. Based on the conducted research, it was observed that samples with a 20% and 40% substitution of animal fat with flaxseed oil had the lowest MUFA content among all experimental samples. In contrast, the samples with rapeseed oil showed the highest MUFA content and lower PUFA levels. This is due to an approximately 10% increase in oleic acid in these samples. Oleic acid is a compound with a beneficial influence on the cardiovascular system and lipid metabolism of the body. In terms of cardiovascular disease prevention, it is recommended to increase the proportion of oleic acid in the diet in preference to saturated acids [38,46,47].

Polyunsaturated fatty acids include linoleic acid (LA, C18:2), representing the n-6 family, and α-linolenic acid (ALA, C18:3), representing the n-3 family. Linoleic acid is found in sunflower, corn, and soybean oils, while α-linolenic acid is found in fish, walnuts, and flaxseed and rapeseed oils. Research from various authors indicates that replacing saturated fatty acids with n-6 polyunsaturated fatty acids reduces the incidence of cardiovascular diseases in the population [42,46,48]. Experimental sausages, such as those made with liver pate, have low ALA content. However, the highest ALA levels among these products were found in samples where animal fat was substituted with flaxseed oil, containing six times more ALA than the samples with rapeseed oil. In contrast, in samples where rapeseed oil was used as a partial substitute for animal fat, the ALA content was, on average, nine times higher than in those with corn or sunflower oil. The study also highlighted the analysis of the n-6 to n-3 fatty acid ratio. The samples with rapeseed oil substitution had the closest ratio to dietary recommendations, ranging from 4.3:1 to 4.4:1.

Too much of a difference between these fatty acids in the diet can disrupt the balance of synthesized eicosanoids, which often act antagonistically, which in turn favors the pathogenesis of various diseases, including cardiovascular diseases, cancers, and inflammatory and autoimmune diseases [49,50]. In the conducted studies, too high a proportion of acids from the n-6 to n-3 family was observed in samples where animal fat was replaced with corn, sunflower, and soybean oils. The highest ratio was found in the samples with corn oil, where a 40% substitution resulted in a ratio of as much as 123:1. In samples with 20% and 40% substitution of animal fat with sunflower oil, the ratios were 74.4:1 and 89.9:1, respectively. These results are three times higher than those obtained in studies conducted by Rodríguez-Carpena et al. [51], which revealed that the proportion of n-6 to n-3 fatty acids in frikadelles produced with an animal fat replaced at 50% with sunflower oil was 20:1.

The progression of autooxidation processes depends on the amount of unsaturated fatty acids and especially polyunsaturated fatty acids in the product. The reaction rate increases with the degree of fat unsaturation [52,53]. Oxidation processes, regardless of their cause, generally lead to the breakdown of triglyceride molecules. Initially, peroxides and hydroperoxides are formed, which then decompose and transform into short-chain compounds. The main products of this decomposition are aldehydes, with smaller amounts of lower fatty acids, alcohols, and, less commonly, ketones. The amount of peroxides, which indicates the degree of fat oxidation, is measured by the peroxide value, which reflects the extent of fat oxidation. In the present study, it was observed that the highest increase in peroxide value during 15 days of storage occurred in experimental sausages where oils high in polyunsaturated fatty acids were used. These were the samples where animal fat was replaced with corn, sunflower, and soybean oils. PUFAs are significantly less oxidatively stable than MUFAs, meaning that they oxidize more quickly and require less activation energy [54,55]. Therefore, products rich in PUFAs (e.g., flaxseed oil, fish oil) are more prone to rancidity compared with products rich in MUFAs (e.g., olive oil, canola oil).

The TBARS content is a standard indicator for assessing oxidative changes occurring in fat. The malondialdehyde detected by this test is one of the secondary products of autoxidation [56,57,58]. The rate of these modifications is determined by many factors, including fatty acid composition, the presence of prooxidants and antioxidants, and storage conditions [58,59,60]. In the tested samples, a statistically significant effect of storage time on the increase in TBARS values was observed. The energy required for the oxidation process in the case of monounsaturated fatty acids (MUFAs) and polyunsaturated fatty acids (PUFAs) differs mainly due to the different number of double bonds in their structure. PUFAs are much less oxidatively stable than MUFAs, which means that they oxidize faster, requiring less activation energy. Therefore, products rich in PUFAs (e.g., linseed and fish oils) are more susceptible to rancidity than products rich in MUFAs (e.g., olive oil, rapeseed oil). Fat oxidation can be limited by, among other things, adding antioxidants, including di-tert-butylhydroxytoluene (BHT), mono-tert-butylhydroxyanisole (BHA), tert-butylhydroquinone (TBHQ) or gallates. However, the safety of using synthetic antioxidants in food is currently being questioned due to the results of toxicological studies [61,62,63,64]. Therefore, compounds naturally occurring in plant raw materials with antioxidant properties are becoming increasingly important. These compounds are considered safer and, above all, are better accepted by consumers. They play an important role in the fight against free radicals, which react with protein, lipid and saccharide molecules and cause their oxidation. A valuable source of antioxidants are spices and herbs and their extracts, e.g., rosemary, sage, oregano, thyme, tea (green, yellow, red).

Similar results were observed Dzudie et al. [65] when testing beef pâtés. In contrast Martin et al. [66], a study that evaluated pâtés made with partial substitution of animal fat by olive oil and/or conjugated linoleic acid reported that TBARS values did not change during 70 days of storage most likely because of the addition of sodium nitrite. In the research, it was demonstrated that samples with partial replacement of animal fat with oils exhibited a lower rate of change in TBARS values compared with the control sample.

Sensory studies allow for the understanding of consumer behaviors and preferences, which are crucial factors in food selection and acceptance. Additionally, the information obtained during sensory studies provides valuable insights for the development and market introduction of new products [67,68,69]. It is also possible to determine whether modification of the product will increase its quality and overall acceptability [69]. The sensory durability of products depends on the condition and type of ingredients used in production, the technological process, the type of packaging employed and the microbiological state of the finished product. These modifications are caused by the physico-chemical and microbiological reactions [70,71] which take place within the product, and their rate depends, among other factors, on the storage temperature. When choosing food products, consumers prioritize quality, which is associated with freshness; the intensity of aroma and taste; appearance; color; price; and the information provided on the label. In the conducted studies, the evaluation panel observed differences in color, aroma, and taste among the tested samples. The type and quantity of oil used had a statistically significant effect on the influence it had on the sensory quality of the final product.

Sodium chloride is one of the additives that are used in sausage production. Its addition increases the water-binding capacity of the protein and improves the emulsifying properties, inhibits the growth of certain microorganisms and is an essential substance for the acceptance of the taste of the meat product [72,73,74,75]. The salt content of meat products typically ranges from 1.8% to 4.0% and is governed by the provisions [76]. The World Health Organization (WHO) [77] recommends a salt intake of <5 g/day/person (2 g sodium) because too much sodium in the diet may increase blood pressure and eventually lead to cardiovascular diseases (CVDs) [74,76,78]. The experimental sausages met the guidelines of the Polish Standard PN-A-82007:1996/Az1:1998 (1998) [35], which specifies that the salt content in offal sausages should not exceed 2%. The sodium chloride content of 1.65 to 1.70% in experimental trials with the substitution of animal fat with oils may be related to the change in the mass structure of the stuffing as a result of heat treatment and the use of semi-permeable casings. It may also result from the type of vegetable oil added as part of the recipe, which is aimed at increasing fat content and which may affect the proportions of other ingredients, including salt. Sensory evaluation of the saltiness intensity of the tested sausages revealed that, regardless of the level of substitution of animal fat with oils, these samples exhibited a stronger salty taste compared with the control sample.

From a physical point of view, the reduction of firmness and shear work in meat batters is associated with the improvement of their spreadability, as it refers to the ability of elasto-viscoplastic materials to deform [3,79]. The reduction in hardness and improvement in spreadability after the addition of oils results from the increased share of low-melting fractions in the fat composition. Melting points of the used oils are as follows: −24 °C for linseed oil, −17 °C for sunflower oil, −16 °C for soybeen oil, −11 °C for corn oil and −10 for rapeseed oil. Wright [80] demonstrated that, as the oil content in the mixture increases, the solid phase content at a given temperature decreases, resulting in reduced hardness and improved spreadability. The studies conducted showed that higher oil content in the pate leads to a lower solid phase content at a given temperature, thus reducing hardness and enhancing spreadability. Similar findings have been reported by Zupanjac et al. [81].

The desirability of the finished product’s taste was also assessed. The taste desirability is the impression perceived by the taste sense under the influence of specific, soluble and chemical stimulus substances [67,69]. The results of studies investigating the possibility of replacing animal fat with vegetable oils have demonstrated a significant impact of this substitution on the sensory desirability of the taste of the evaluated products. It was found that the samples with partial replacement of animal fat by rapeseed oil had the most desirable taste among the other tested sausages.

Baek et al. [82] found that 20% substitution of pork fat with rapeseed oil did not result in any significant changes in terms of color in cross-section, smell or overall desirability compared with the control sample. However, there was a significant reduction in the sensory quality of the produced sample in which 20% of pork fat was substituted with linseed oil. In the conducted experiment, the type and amount of animal fat replaced with oil had a statistically significant impact on the sensory evaluation of the tested parameters. The study showed that the samples with rapeseed oil received the highest ratings among the evaluated offal sausages.

## 5. Conclusions

The research showed that replacing part of the animal fat with vegetable oils such as flaxseed, corn, rapeseed, soybean, and sunflower in the recipe for liver pâté-type offal sausages allows one to obtain products with high sensory attractiveness and antioxidant stability. It was demonstrated that, among the tested sausages, the samples with a 20% content of rapeseed oil exhibited the best oxidative stability properties and the most favorable fatty acid ratio of n-6 to n-3, one which is close to nutritional recommendations. In these sausages, the content of α-linolenic acid was, on average, eight times higher compared with the samples with corn or sunflower oil.

The sensory evaluation results confirm that the sample with a 20% substitution of animal fat for rapeseed oil, after 15 days of storage, had a more stable and desirable color on the cut surface, aroma, saltiness, and taste compared with the other experimental liver pâté.

The incorporation of vegetable oils into liver pâté-style offal sausages presents a promising alternative for creating healthier meat products that align with industry standards and modern consumer preferences. However, further research is required to optimize this substitution and enhance its effectiveness.

## Figures and Tables

**Table 1:** Composition of liver sausage [g kg^−1^].

Oil	% of Replacement	Ingredients [g/kg]				
		Pork Meat (Class II)	Pork Fatback	Oil	Pork Liver	Mix of Spices
Control sample (Test without oil)	0	430	420	-	150	21
Corn oil (CO)	20	430	336	84	150	21
	40	430	252	168	150	21
Linseed oil (LO)	20	430	336	84	150	21
	40	430	252	168	150	21
Rapeseed oil (RO)	20	430	336	84	150	21
	40	430	252	168	150	21
Sunflower oil (SunO)	20	430	336	84	150	21
	40	430	252	168	150	21
Soybean oil (SO)	20	430	336	84	150	21
	40	430	252	168	150	21

**Table 2:** The composition and mutual proportions of the individual fatty acid groups in the edible fats examined $\bar{x}$ (*n* = 6) ± sd.

Fatty Acids	Types of Edible Fats					
	Animal Fat	Corn Oil (CO)	Linseed Oil (LO)	Rapeseed Oil (RO)	Sunflower Oil (Suno)	Soybean Oil (SO)
C12:0	0.12 ± 0.02	0.00	0.00	0.00	0.00	0.00
C14:0	1.80 ± 0.02	0.00	0.00	0.00	0.00	0.00
C16:0	25.89 ^f^± 0.08	12.50 ^e^ ± 0.02	5.82 ^c^ ± 0.07	4.44 ^b^ ± 0.02	6.34 ^d^ ± 0.04	10.62 ^a^ ± 0.01
C16:1	2.73 ^c^ ± 0.04	0.00	0.17 ^b^ ± 0.00	0.20 ^b^ ± 0.00	0.07 ^a^ ± 0.00	0.09 ^a^ ± 0.00
C17:0	0.00	0.05 ^a^ ± 0.04	0.00	0.00	0.00	0.10 ^b^ ± 0.00
C18:0	13.98 ^f^ ± 0.05	2.90 ^b^ ± 0.02	3.83 ^e^ ± 0.05	1.58 ^a^ ± 0.02	3.35 ^c^ ± 0.05	3.53 ^d^ ± 0.03
C18:1	45.91 ^e^± 0.08	27.55 ^d^ ± 0.14	21.66 ^a^ ± 0.23	61.95 ± 0.12	24.23 ^c^ ± 0.13	23.54 ^b^ ± 0.17
C18:2	7.63 ^a^ ± 0.04	55.65 ^d^ ± 1.05	16.12 ^b^ ± 0.15	19.26 ^c^ ± 0.01	63.91 ^f^ ± 0.15	56.84 ^e^ ± 0.09
C18:3	1.61 ^b^ ± 0.02	1.10 ^b^ ± 0.01	51.82 ^e^ ± 1.11	11.30 ^d^ ± 0.00	0.33 ^a^ ± 0.00	4.05 ^c^ ± 0.07
C20:0	0.19 ^a^ ± 0.01	0.25 ^b^ ± 0.00	0.25 ^b^ ± 0.00	0.55 ^d^ ± 0.06	0.21 ^a^ ± 0.00	0.40 ^c^ ± 0.00
C20:1	0.00	0.00	0.33 ^d^ ± 0.01	0.21 ^b^ ± 0.02	0.16 ^a^ ± 0.00	0.24 ^c^ ± 0.00
C22:0	0.00	0.00	0.00	0.32 ^a^ ± 0.01	0.81 ^c^ ± 0.01	0.45 ^b^ ± 0.01
C22:1	0.17 ^a^ ± 0.01	0.00	0.00	0.21 ^b^ ± 0.00	0.00	0.00
C24:0	0.00	0.00	0.00	0.00	0.00	0.15 ± 0.00
∑SFA	41.96 ^e^ ± 11.36	15.70 ^d^ ± 4.97	9.90 ^b^ ± 2.54	6.88 ^a^ ± 1.71	10.70 ^c^ ± 2.57	15.25 ^d^ ± 4.17
∑MUFA	48.81 ^e^ ± 25.70	27.55 ^d^ ± 13.85	22.16 ^a^ ± 10.75	62.56 ^f^ ± 30.87	25.07 ^c^ ± 12.37	23.87 ^b^ ± 11.71
∑PUFA	9.24 ^a^ ± 4.26	56.75 ^c^ ± 38.57	67.94 ^f^ ± 25.24	30.56 ^b^ ± 5.62	64.24 ^e^ ± 44.95	60.88 ^d^ ± 37.25
PUFA/SFA	0.22	3.61	6.86	4.44	6.00	3.99
n-6/n-3	4.75:1	50.59:1	0.31:1	1.70:1	191.46:1	14.05:1

Explanations: ^a,b…^—mean values ± standard deviation followed by different letters in rows refer to statistically significant differences between the types of samples (*p* ≤ 0.05); $\bar{x}$—mean value; *n*—number of replications; sd—standard deviation; ∑SFA—total saturated fatty acids; ∑MUFA—total monounsaturated fatty acids; ∑PUFA—total polyunsaturated fatty acids; n-6/n-3—PUFA n-6/PUFA n-3 ratio.

**Table 3:** The content and mutual proportions of individual groups of fatty acids in experimental sausages $\bar{x}$ (*n* = 6) ± sd.

Fatty Acids	Control Sample	Percentage of Animal Fat Replaced with Vegetable Oil									
		Corn Oil		Linseed Oil		Rapeseed Oil		Sunflower Oil		Soybean Oil	
		20	40	20	40	20	40	20	40	20	40
*∑*SFA	39.83 ^j^ ± 10.38	38.54 ^i^ ± 9.98	34.28 ^c^ ± 8.94	35.55 ^e^ ± 9.22	33.19 ^b^ ± 8.58	37.05 ^f^ ± 9.57	34.70 ^d^ ± 9.11	37.71 ^g^ ± 9.68	32.66 ^a^ ± 8.28	37.83 ^h^ ± 9.66	34.65 ^d^ ± 8.77
*∑*MUFA	46.69 ^f^ ± 18.41	45.59 ^c^ ± 18.01	45.83 ^d^ ± 18.41	41.88 ^a^± 16.99	43.65 ^b^ ± 17.49	52.40 ^h^ ± 21.14	54.42 ^i^ ± 21.83	46.44 ^ef^ ± 18.38	46.42 ^e^ ± 18.92	46.90 ^g^ ± 18.55	46.30 ^e^ ± 18.67
∑PUFA	13.48 ^c^ ± 5.64	15.87 ^e^ ± 7.83	19.89 ^g^ ± 9.84	22.57 ^i^ ± 6.47	23.16 ^j^ ± 6.65	10.55 ^a^ ± 3.62	10.95 ^b^ ± 3.75	15.85 ^e^ ± 7.78	20.92 ^h^ ± 10.30	15.27 ^d^ ± 6.88	19.05 ^f^ ± 8.76
PUFA/SFA	0.34	0.41	0.58	0.63	0.70	0.28	0.31	0.42	0.64	0.40	0.55

Explanations: ^a,b…^—mean values ± standard deviation followed by different letters in rows refer to statistically significant differences between the types of samples (*p* ≤ 0.05); $\bar{x}$—mean value; *n*—number of replications; sd—standard deviation; *∑*SFA—total saturated fatty acids; ∑MUFA—total monounsaturated fatty acids; *∑*PUFA—total polyunsaturated fatty acids; n-6/n-3—PUFA n-6/PUFA n-3 ratio.

**Table 4:** The composition of fatty acids from the n-6 and n-3 family in experimental sausages $\bar{x}$ (*n* = 6) ± sd.

Fatty Acids	Control Sample	Percentage of Animal Fat Replaced with Vegetable Oil									
		Corn Oil		Linseed Oil		Rapeseed Oil		Sunflower Oil		Soybean Oil	
		20	40	20	40	20	40	20	40	20	40
C18:2	11.80 ^e^ ± 0.87	15.71 ^g^ ± 0.94	19.73 ^i^ ± 1.02	10.49 ^c^ ± 1.09	10.80 ^d^ ± 0.76	7.96 ^a^ ± 0.07	8.25 ^b^ ± 0.67	15.63 ^g^ ± 0.59	20.68 ^j^ ± 1.01	14.11 ^f^ ± 0.82	17.88 ^h^ ± 0.46
C18:3	1.16 ^c^ ± 0.02	0.16 ^a^ ± 0.00	0.16 ^a^ ± 0.01	11.96 ^e^ ± 0.97	12.26 ^f^ ± 0.65	1.85 ^d^ ± 0.45	1.88 ^d^± 0.23	0.21 ^b^ ± 0.00	0.23 ^b^ ± 0.00	1.16 ^c^ ± 0.04	1.17 ^c^ ± 0.07
n6/n3	10.2:1	98.2:1	123.3:1	0.9:1	0.9:1	4.3:1	4.4:1	74.4:1	89.9:1	12.2:1	15.3:1

Explanations: ^a,b…^—mean values ± standard deviation followed by different letters in rows refer to statistically significant differences between the types of samples (*p* ≤ 0.05); $\bar{x}$—mean value; *n*—number of replications; sd—standard deviation; ∑SFA—total saturated fatty acids; ∑MUFA—total monounsaturated fatty acids; ∑PUFA—total polyunsaturated fatty acids; n-6/n-3—PUFA n-6/PUFA n-3 ratio.

**Table 5:** Changes in peroxide value in the experimental pates during storage (mEqO_2_/kg of sample), $\bar{x}$ (*n* = 6) ± sd (LSD A = 0.01, LSD B = 0.02).

Storage Time (Days)	Control Sample	Corn Oil (CO)		Linseed Oil (LO)		Rapeseed Oil (RO)		Sunflower Oil (Suno)		Soybean Oil (SO)	
		Percentage of Animal Fat Replaced with Vegetable Oil									
		20	40	20	40	20	40	20	40	20	40
1	0.23 ^cA^ ± 0.01	0.22 ^cA^± 0.01	0.24 ^cA^ ± 0.04	0.31 ^hA^ ± 0.08	0.38 ^iA^ ± 0.01	0.18 ^aA^ ± 0.01	0.20 ^bA^ ± 0.02	0.25 ^dA^ ± 0.01	0.30 ^fgA^ ± 0.01	0.27 ^eA^ ± 0.01	0.29 ^fA^ ± 0.03
5	0.34 ^efC^ ± 0.02	0.27 ^aB^ ± 0.01	0.31 ^bcB^ ± 0.02	0.44 ^gB^ ± 0.04	0.62 ^hB^ ± 0.02	0.26 ^aB^ ± 0.03	0.27 ^aB^ ± 0.03	0.33 ^deB^ ± 0.02	0.35 ^fB^ ± 0.01	0.30 ^bB^ ± 0.03	0.32 ^cdB^ ± 0.02
8	0.42 ^eD^ ± 0.01	0.30 ^aC^ ± 0.02	0.34 ^bC^ ± 0.01	0.61 ^hC^ ± 0.03	0.60 ^hB^ ± 0.01	0.40 ^dC^ ± 0.02	0.49 ^fD^ ± 0.06	0.37 ^cC^ ± 0.03	0.52 ^gC^ ± 0.03	0.52 ^gC^ ± 0.04	0.51 ^gC^ ± 0.01
12	0.42 ^aD^ ± 0.04	0.48 ^cD^ ± 0.04	0.60 ^fD^ ± 0.02	0.72 ^gD^ ± 0.04	0.73 ^gC^ ± 0.04	0.48 ^cD^ ± 0.01	0.56 ^dE^ ± 0.05	0.45 ^bD^ ± 0.02	0.61 ^fD^ ± 0.01	0.58 ^eD^ ± 0.02	0.57 ^deD^ ± 0.01
15	0.29 ^aB^ ± 0.02	0.53 ^dE^ ± 0.05	0.65 ^gE^ ± 0.02	0.76 ^iE^ ± 0.03	0.81 ^jD^ ± 0.02	0.40 ^bC^ ± 0.04	0.43 ^cC^ ± 0.04	0.57 ^eE^ ± 0.03	0.69 ^hE^ ± 0.02	0.59 ^fD^ ± 0.06	0.77 ^iE^ ± 0.04
coeff. A × 10^−3^/24 h	6	23	32	34	28	19	22	22	29	27	34
R^2^	0.17	0.93	0.92	0.97	0.92	0.76	0.62	0.97	0.96	0.87	0.93

Explanations: $\bar{x}$—mean value; *n*—number of replications; sd—standard deviation; LSD A—the least significant difference for the type of sample; LSD B—the least significant difference for the time of storage; ^a,b…^—mean values ± standard deviation followed by different letters in rows refer to statistically significant differences between the types of samples (*p* ≤ 0.05); ^A,B…^—mean values ± standard deviation followed by different letters in columns refer to statistically significant differences in the time of storage (*p* ≤ 0.05); linear regression equation: y = Ax + B (y—dependent variable, x—independent variable, A—independent variable coefficient per slope of the line, B—intercept); coefficient A/24 h—modification of coefficient A during 24 h storage; R^2^– coefficient of determination, *p* < 0.05.

**Table 6:** Changes in TBARS values in the experimental pates during storage (mg MDA/kg of the sample) $\bar{x}$ (*n* = 6) ± sd (LSD A = 0.03, LSD B = 0.04).

Storage Time (Days)	Control Sample	Corn Oil (CO)		Linseed Oil (LO)		Rapeseed Oil (RO)		Sunflower Oil (Suno)		Soybean Oil (SO)	
		Percentage of Animal Fat Replaced with Vegetable Oil									
		20	40	20	40	20	40	20	40	20	40
1	1.99 ^fA^ ± 0.03	1.40 ^cA^ ± 0.01	1.26 ^bA^ ± 0.04	1.83 ^dA^ ± 0.09	1.88 ^eA^ ± 0.04	1.20 ^aA^ ± 0.03	1.18 ^aA^ ± 0.07	1.27 ^bA^ ± 0.08	1.27 ^bA^ ± 0.03	1.84 ^dA^ ± 0.06	1.81 ^dA^ ± 0.07
5	2.13 ^hB^ ± 0.06	1.56 ^dB^ ± 0.04	1.53 ^cdB^ ± 0.04	1.95 ^fB^ ± 0.11	2.04 ^gB^ ± 0.07	1.51 ^cB^ ± 0.02	1.51 ^cB^ ± 0.03	1.31 ^aA^ ± 0.05	1.36 ^bB^ ± 0.03	1.77 ^eB^ ± 0.04	1.80 ^eB^ ± 0.01
8	2.51 ^gC^ ± 0.10	2.21 ^dC^ ± 0.05	2.18 ^dC^ ± 0.02	2.74 ^hC^ ± 0.15	2.49 ^fC^ ± 0.06	1.92 ^aC^ ± 0.02	1.95 ^aC^ ± 0.02	2.14 ^cB^ ± 0.06	2.05 ^bC^ ± 0.05	2.31 ^eC^ ± 0.02	2.20 ^dC^ ± 0.01
12	2.66 ^fD^ ± 0.03	2.38 ^deD^ ± 0.05	2.35 ^dD^ ± 0.02	2.89 ^hD^ ± 0.06	2.81 ^gD^ ± 0.05	2.02 ^aD^ ± 0.05	2.29 ^cD^ ± 0.05	2.33 ^dC^ ± 0.09	2.30 ^cD^ ± 0.02	2.41 ^eD^ ± 0.17	2.24 ^bC^ ± 0.04
15	2.52 ^fC^ ± 0.07	2.33 ^dD^ ± 0.03	2.33 ^dD^ ± 0.04	2.86 ^gD^ ± 0.05	2.88 ^gE^ ± 0.04	1.99 ^aD^ ± 0.09	2.13 ^bE^ ± 0.03	2.33 ^dC^ ± 0.17	2.30 ^dD^ ± 0.01	2.41 ^eD^ ± 0.02	2.22 ^cC^ ± 0.05
coeff. A × 10^−3^/24 h	45	76	85	86	79	60	77	89	85	50	36
R^2^	0.77	0.84	0.87	0.83	0.95	0.85	0.87	0.83	0.87	0.77	0.75

Explanations: $\bar{x}$—mean value; *n*—number of replications; sd—standard deviation; LSD A—the least significant difference for the type of sample; LSD B—the least significant difference for the time of storage; ^a,b…^—mean values ± standard deviation followed by different letters in rows refer to statistically significant differences between the types of samples (*p* ≤ 0.05); ^A,B…^—mean values ± standard deviation followed by different letters in columns refer to statistically significant differences in the time of storage (*p* ≤ 0.05); linear regression equation: y = Ax + B (y—dependent variable, x—independent variable, A—independent variable coefficient per slope of the line, B—intercept); coefficient A/24 h—modification of coefficient A during 24 h storage; R^2^– coefficient of determination, *p* < 0.05.

**Table 7:** Textural properties of experimental pâtés $\bar{x}$ (*n* = 4) ± sd.

Sample	Percentage of Animal Fat Replaced with Vegetable Oil	Firmness [N]	Work of Shear [N*s]	Stickiness [N]	Adhesion [N*s]
Control sample	0	27.273 ^f^ ± 1.938	47.381 ^d^ ± 3.539	−28.370 ^e^ ± 1.165	−2.805 ^cd^ ± 0.355
Corn oil (CO)	20	14.701 ^bd^ ± 0.903	25.003 ^a^ ± 0.822	−14.563 ^a^ ± 1.665	−1.837 ^ab^ ± 0.211
	40	8.486 ^a^ ± 0.090	15.192 ^bc^ ± 0.092	−10.673 ^b^ ± 0.268	−2.039 ^a^ ± 0.030
Linseed oil (LO)	20	14.288 ^bd^ ± 0.757	24.161 ^a^ ± 1.587	−13.807 ^ac^ ± 1.946	−1.695 ^ab^ ± 0.313
	40	8.353 ^a^ ± 0.459	12.813 ^c^ ± 0.460	−6.102 ^f^ ± 0.342	−1.345 ^b^ ± 0.086
Rapeseed oil (RO)	20	15.903 ^d^ ± 1.554	25.743 ^a^ ± 0.645	−15.377 ^ac^ ± 1.507	−1.879 ^a^ ± 0.159
	40	10.992 ^ce^ ± 0.965	17.223 ^b^ ± 0.751	−10.349 ^b^ ± 0.429	−1.828 ^ab^ ± 1.828
Sunflower oil (SunO)	20	9.971 ^ac^ ± 0.470	17.877 ^b^ ± 1.211	−10.908 ^b^ ± 0.160	−1.652 ^ab^ ± 0.116
	40	9.395 ^ac^ ± 0.383	14.734 ^bc^ ± 0.276	−11.562 ^bd^ ± 0.424	−3.322 ^d^ ± 0.073
Soybean oil (SO)	20	13.348 ^b^ ± 0.205	24.568 ^a^ ± 0.607	−17.078 ^c^ ± 0.450	−2.702 ^c^ ± 0.072
	40	13.053 ^be^ ± 0.091	22.579 ^a^ ± 0.774	−16.061 ^ac^ ± 0.359	−2.770 ^c^ ± 0.390

Explanations: ^a,b…^—mean values ± standard deviation followed by different letters in rows refer to statistically significant differences between the types of samples (*p* ≤ 0.05); $\bar{x}$—mean value; *n*—number of replications; sd—standard deviation.

## Data Availability

The original contributions presented in this study are included in the article. Further inquiries can be directed to the corresponding author.

## References

[1] Estévez M., Cava R. (2004). Lipid and protein oxidation, release of iron from heme molecule and colour deterioration during refrigerated storage of liver pâté. Meat Sci..

[2] Estévez M., Ramírez R., Ventanas S., Cava R. (2007). Sage and rosemary essential oils versus BHT for the inhibition of lipid oxidative reactions in liver pâté. LWT-Food Sci. Technol..

[3] Terrasa A.M., Dello Staffolo M., Tomás M.C. (2016). Nutritional improvement and physicochemical evaluation of liver pâté formulations. LWT.

[4] Domínguez R., Pateiro M., Gagaoua M., Barba F.J., Zhang W., Lorenzo J.M. (2019). A comprehensive review on lipid oxidation in meat and meat products. Antioxidants.

[5] Ganhão R., Morcuende D., Estévez M. (2010). Protein oxidation in emulsified cooked burger patties with added fruit extracts: Influence on colour and texture deterioration during chill storage. Meat Sci..

[6] Choi Y.S., Park K.S., Kim H.W., Hwang K.E., Song D.H., Choi M.S., Lee S.Y., Paik H.D., Kim C.J. (2013). Quality characteristics of reduced-fat frankfurters with pork fat replaced by sunflower seed oils and dietary fiber extracted from makgeolli lees. Meat Sci..

[7] Delgado-Pando G., Cofrades S., Rodríguez-Salas L., Jiménez-Colmenero F. (2011). A healthier oil combination and konjac gel as functional ingredients in low-fat pork liver pâté. Meat Sci..

[8] Domínguez R., Pateiro M., Sichetti Munekata P.E., Bastianello Campagnol P.C., Lorenzo J.M. (2017). Influence of partial pork backfat replacement by fish oil on nutritional and technological properties of liver pâté. Eur. J. Lipid Sci. Technol..

[9] Gómez I., Janardhanan R., Ibañez F.C., Beriain M.J. (2020). The effects of processing and preservation technologies on meat quality: Sensory and nutritional aspects. Foods.

[10] Muguerza E., Gimeno O., Ansorena D., Astiasarán I. (2004). New formulations for healthier dry fermented sausages:a review. Trends Food Sci. Technol..

[11] Shen J., Liu Y., Wang X., Bai J., Lin L., Luo F., Zhong H. (2023). A Comprehensive review of health-benefiting components in rapeseed oil. Nutrients.

[12] Orsavova J., Misurcova L., Vavra Ambrozova J., Vicha R., Mlcek J. (2015). Fatty acids composition of vegetable oils and its contribution to dietary energy intake and dependence of cardiovascular mortality on dietary intake of fatty acids. Int. J. Mol. Sci..

[13] Maszewska M., Florowska A., Dłuzewska E., Wroniak M., Marciniak-Lukasiak K., Zbikowska A. (2018). Oxidative stability of selected edible oils. Molecules.

[14] Tripathi V., Abidi A.B., Marker S., Bilal S. (2013). Linseed and linseed oil: Health benefits-a review. Int. J. Pharm. Biol. Sci..

[15] Szterk A., Roszko M., Sosińska E., Derewiaka D., Lewicki P.P. (2010). Chemical composition and oxidative stability of selected plant oils. J. Am. Oil Chem. Soc..

[16] Bukowski M.R., Goslee S. (2024). Climate-based variability in the essential fatty acid composition of soybean oil. Am. J. Clin. Nutr..

[17] Makni M., Haddar A., Fraj A.B., Zeghal N. (2015). Physico-chemical properties, composition, and oxidative stability of olive and soybean oils under different conditions. Int. J. Food Prop..

[18] Maki K.C., Hasse W., Dicklin M.R., Bell M., Buggia M.A., Cassens M.E., Eren F. (2018). Corn Oil Lowers Plasma Cholesterol Compared with Coconut Oil in Adults with Above-Desirable Levels of Cholesterol in a Randomized Crossover Trial. J. Nutr..

[19] Maki K.C., Lawless A.L., Kelley K.M., Kaden V.N., Geiger C.J., Dicklin M.R. (2015). Corn oil improves the plasma lipoprotein lipid profile compared with extra-virgin olive oil consumption in men and women with elevated cholesterol: Results from a randomized controlled feeding trial. J. Clin. Lipidol..

[20] Susik J. (2021). Corn oil production methods determining its chemical properties (in Polish). Zywn. Nauk. Technol. Jakosc/Food Sci. Technol. Qual..

[21] Kozłowska M., Gruczyńska E. (2018). Comparison of the oxidative stability of soybean and sunflower oils enriched with herbal plant extracts. Chem. Pap..

[22] Gotor A.A., Rhazi L. (2016). Effects of refining process on sunflower oil minor components: A review. OCL-Oilseeds Fats Crops Lipids.

[23] Regitano Neto A., Miguel A.M.R.d.O., Mourad A.L., Henriques E.A., Alves R.M.V. (2016). Environmental effect on sunflower oil quality. Crop Breed. Appl. Biotechnol..

[24] Rai A., Mohanty B., Bhargava R. (2016). Supercritical extraction of sunflower oil: A central composite design for extraction variables. Food Chem..

[25] (1997). Methods of Test for Meat and Meat Products. Determination of Moisture Content.

[26] (2009). Animal Feeding Stuffs. Determination of Nitrogen Content and Calculation of Crude Protein Content. Part 2: Block Digestion/Steam Distillation Method.

[27] (2017). Animal and Vegetable Fats and Oils. Determination of Peroxide Value. Iodometric (Visual) Endpoint Determination.

[28] (1996). Meat and Meat Products-Determination of Chloride Content-Part 1: Volhard Method.

[29] Folch J., Lees M., Stanley G.H.S. (1957). A simple method for the isolation and purification of total lipids from animal tissues. J. Biol. Chem..

[30] AOCS (2017). AOCS Official Method Ce 1h-05 determination of cis-, trans-, saturated, monounsaturated and polyunsaturated fatty acids in vegetable or non-ruminant animal oils and fats by capillary GLC. Methods Recomm Pract AOCS.

[31] Tarladgis B.G., Watts B.M., Younathan M.T., Dugan L. (1960). A distillation method for the quantitative determination of malonaldehyde in rancid foods. J. Am. Oil Chem. Soc..

[32] Pikul J., Leszczynski D.E., Kummerow F.A. (1989). Evaluation of three modified TBA methods for measuring lipid oxidation in chicken meat. J. Agric. Food Chem..

[33] (2016). Analiza Sensoryczna-Metodyka-Ogólne Wytyczne Ustalania Profilu Sensorycznego.

[34] (2009). Analiza Sensoryczna-Terminologia.

[35] (1998). Przetwory Mięsne-Wędliny.

[36] Fadda A., Sanna D., Sakar E.H., Gharby S., Mulas M., Medda S., Yesilcubuk N.S., Karaca A.C., Gozukirmizi C.K., Lucarini M. (2022). Innovative and Sustainable Technologies to Enhance the Oxidative Stability of Vegetable Oils. Sustainability.

[37] Lorenzo J.M., Pateiro M., Fontán M.C.G., Carballo J. (2014). Effect of fat content on physical, microbial, lipid and protein changes during chill storage of foal liver pâté. Food Chem..

[38] Shramko V.S., Polonskaya Y.V., Kashtanova E.V., Stakhneva E.M., Ragino Y.I. (2020). The short overview on the relevance of fatty acids for human cardiovascular disorders. Biomolecules.

[39] Mińkowski K., Grześkiewicz S., Jerzewska M. (2011). Assessment of nutritive value of plant oils with high content of linolenic acids based on the composition of fatty acids, tocopherols, and sterols. Zywn. Nauk. Technol. Jakosc/Food Sci. Technol. Qual..

[40] Awogbemi O., Onuh E.I., Inambao F.L. (2019). Comparative study of properties and fatty acid composition of some neat vegetable oils and waste cooking oils. Int. J. Low-Carbon Technol..

[41] Makała H., Duda-Chodak A., Najgebauer-Lejko D., Drożdż I., Tarko T. (2016). The influence of the level of plant oil additives in model ground meat products on the dynamics of oxidative transformations. The Role of Technological Processes in Shaping Food Quality.

[42] Mariamenatu A.H., Abdu E.M. (2021). Overconsumption of Omega-6 Polyunsaturated Fatty Acids (PUFAs) versus Deficiency of Omega-3 PUFAs in Modern-Day Diets: The Disturbing Factor for Their “Balanced Antagonistic Metabolic Functions” in the Human Body. J. Lipids.

[43] Ponnampalam E.N., Sinclair A.J., Holman B.W.B. (2021). The sources, synthesis and biological actions of omega-3 and omega-6 fatty acids in red meat: An overview. Foods.

[44] Jiménez-Colmenero F. (2007). Healthier lipid formulation approaches in meat-based functional foods. Technological options for replacement of meat fats by non-meat fats. Trends Food Sci. Technol..

[45] Kris-Etherton P.M., Grieger J.A., Etherton T.D. (2009). Dietary reference intakes for DHA and EPA. Prostaglandins Leukot. Essent. Fat. Acids.

[46] Briggs M.A., Petersen K.S., Kris-Etherton P.M. (2017). Saturated fatty acids and cardiovascular disease: Replacements for saturated fat to reduce cardiovascular risk. Healthc..

[47] Piccinin E., Cariello M., De Santis S., Ducheix S., Sabbà C., Ntambi J.M., Moschetta A. (2019). Role of oleic acid in the gut-liver axis: From diet to the regulation of its synthesis via Stearoyl-CoA desaturase 1 (SCD1). Nutrients.

[48] Wang D.D. (2018). Dietary n-6 polyunsaturated fatty acids and cardiovascular disease: Epidemiologic evidence. Prostaglandins Leukot. Essent. Fat. Acids.

[49] Huerta-Yépez S., Tirado-Rodriguez A.B., Hankinson O. (2016). Role of diets rich in omega-3 and omega-6 in the development of cancer. Bol. Med. Hosp. Infant. Mex..

[50] Mińkowski K., Grześkiewicz S., Jerzewska M., Ropelewska M. (2010). Chemical composition profile of plant oils with high content of linolenic acids. Zywn. Nauka Technol. Jakosc.

[51] Rodríguez-Carpena J.G., Morcuende D., Estévez M. (2012). Avocado, sunflower and olive oils as replacers of pork back-fat in burger patties: Effect on lipid composition, oxidative stability and quality traits. Meat Sci..

[52] Ahmed M., Pickova J., Ahmad T., Liaquat M., Farid A., Jahangir M. (2016). Oxidation of Lipids in Foods. Sarhad J. Agric..

[53] Kumar Y., Yadav D.N., Ahmad T., Narsaiah K. (2015). Recent trends in the use of natural antioxidants for meat and meat products. Compr. Rev. Food Sci. Food Saf..

[54] Saini R.K., Prasad P., Sreedhar R.V., Akhilender Naidu K., Shang X., Keum Y.-S. (2021). Omega−3 Polyunsaturated Fatty Acids (PUFAs): Emerging Plant and Microbial Sources, Oxidative Stability, Bioavailability, and Health Benefits—A Review. Antioxidants.

[55] Saini R.K., Keum Y.S. (2018). Omega-3 and omega-6 polyunsaturated fatty acids: Dietary sources, metabolism, and significance—A review. Life Sci..

[56] Ayala A., Muñoz M.F., Argüelles S. (2014). Lipid peroxidation: Production, metabolism, and signaling mechanisms of malondialdehyde and 4-hydroxy-2-nonenal. Oxid. Med. Cell. Longev..

[57] Bilska A., Kowalski R., Kalinowska A. (2014). Changes of the lipids fraction in liver sausage with the addition of oil. Med. Weter..

[58] Wenjiao F., Yongkui Z., Yunchuan C., Junxiu S., Yuwen Y. (2014). TBARS predictive models of pork sausages stored at different temperatures. Meat Sci..

[59] Bilska A., Waszkowiak K., Błaszyk M., Rudzińska M., Kowalski R. (2018). Effect of liver pâté enrichment with flaxseed oil and flaxseed extract on lipid composition and stability. J. Sci. Food Agric..

[60] Kahraman T., Issa G., Bingol E.B., Kahraman B.B., Dumen E. (2015). Effect of rosemary essential oil and modified-atmosphere packaging (MAP) on meat quality and survival of pathogens in poultry fillets. Brazilian J. Microbiol..

[61] Gahruie H.H., Hosseini S.M.H., Taghavifard M.H., Eskandari M.H., Golmakani M.-T., Shad E. (2017). Lipid oxidation, color changes, and microbiological quality of frozen beef burgers incorporated with shirazi thyme, cinnamon, and rosemary extracts. J. Food Qual..

[62] Bianchin M., Pereira D., dos Reis S.A., Almeida J.D.F., do Silva L.D., de Moura C., Carpes S.T. (2017). Rosemary essential oil and lyophilized extract as natural antioxidant source to prevent lipid oxidation in pork sausage. Adv. J. Food Sci. Technol..

[63] Shahidi F., Ambigaipalan P. (2015). Phenolics and polyphenolics in foods, beverages and spices: Antioxidant activity and health effects-A review. J. Funct. Foods.

[64] Xu L., Zhu M.-J., Liu X.-M., Cheng J.-R. (2018). Inhibitory effect of mulberry (*Morus alba*) polyphenol on the lipid and protein oxidation of dried minced pork slices during heat processing and storage. LWT-Food Sci. Technol..

[65] Dzudie T., Kouebou C.P., Essia-Ngang J.J., Mbofung C.M.F. (2004). Lipid sources and essential oils effects on quality and stability of beef patties. J. Food Eng..

[66] Martin D., Ruiz J., Kivikari R., Puolanne E. (2008). Partial replacement of pork fat by conjugated linoleic acid and/or olive oil in liver pâtés: Effect on physicochemical characteristics and oxidative stability. Meat Sci..

[67] Boesveldt S., Bobowski N., McCrickerd K., Maître I., Sulmont-Rossé C., Forde C.G. (2018). The changing role of the senses in food choice and food intake across the lifespan. Food Qual. Prefer..

[68] Ruiz-Capillas C., Herrero A.M. (2021). Sensory analysis and consumer research in new product development. Foods.

[69] Sharif M.K., Butt M.S., Sharif H.R., Nasir M. (2017). Sensory Evaluation and Consumer Research. Handbook of Food Science and Technology.

[70] Doulgeraki A.I., Ercolini D., Villani F., Nychas G.J.E. (2012). Spoilage microbiota associated to the storage of raw meat in different conditions. Int. J. Food Microbiol..

[71] Nychas G.J.E., Skandamis P.N., Tassou C.C., Koutsoumanis K.P. (2008). Meat spoilage during distribution. Meat Sci..

[72] Kim T.K., Yong H.I., Jung S., Kim H.W., Choi Y.S. (2021). Effect of reducing sodium chloride based on the sensory properties of meat products and the improvement strategies employed: A review. J. Anim. Sci. Technol..

[73] Kim T.K., Yong H.I., Jung S., Kim H.W., Choi Y.S. (2021). Technologies for the production of meat products with a low sodium chloride content and improved quality characteristics—A review. Foods.

[74] Kurćubić V., Stajić S., Miletić N., Stanišić N. (2022). Healthier Meat Products Are Fashionable—Consumers Love Fashion. Appl. Sci..

[75] Olmedilla-Alonso B., Jiménez-Colmenero F., Sánchez-Muniz F.J. (2013). Development and assessment of healthy properties of meat and meat products designed as functional foods. Meat Sci..

[76] Grasso S., Brunton N.P., Lyng J.G., Lalor F., Monahan F.J. (2014). Healthy processed meat products-Regulatory, reformulation and consumer challenges. Trends Food Sci. Technol..

[77] World Health Organization, Food and Agriculture Organization UN (2003). Diet, Nutrition and the Prevention of Chronic Diseases: Report of a Joint WHO/FAO Expert Consultation 2002.

[78] Desmond E. (2006). Reducing salt: A challenge for the meat industry. Meat Sci..

[79] Rezler R., Krzywdzińska-Bartkowiak M., Piątek M. (2021). The influence of the substitution of fat with modified starch on the quality of pork liver pâtés. LWT.

[80] Wright A.J., Scanlon M.G., Hartel R.W., Marangoni A.G. (2001). Rheological Properties of Milkfat and Butter. J. Food Sci..

[81] Županjac M., Ikonić P., Šojić B., Đermanović B. (2023). Physicochemical and sensory properties of pork liver pâté formulated with sunflower oleogel as fat substituent. Meat Technol..

[82] Baek K.H., Utama D.T., Lee S.G., An B.K., Lee S.K. (2016). Effects of replacing pork back fat with canola and flaxseed oils on physicochemical properties of emulsion sausages from spent layer meat. Asian-Australas. J. Anim. Sci..

